# Comparison of the efficacy of unilateral versus bilateral pedicle approach vertebroplasty in the treatment of osteoporotic thoracic and lumbar vertebral compression fractures: A retrospective cohort study

**DOI:** 10.1097/MD.0000000000043632

**Published:** 2025-08-01

**Authors:** Zhiyu Liu, Haigang Zhang, Xiaoliang Ma, Weiliang Zhang, Bin Shi

**Affiliations:** a Xi’an People’s Hospital (Xi’an Fourth Hospital), Xi’an, Shaanxi Province, China.

**Keywords:** bilateral pedicle approach, osteoporotic vertebral compression fractures, percutaneous kyphoplasty, thoracolumbar spine, unilateral pedicle approach

## Abstract

Osteoporotic vertebral compression fractures (OVCFs) are prevalent among the elderly and significantly impact quality of life. Percutaneous kyphoplasty (PKP) offers effective, minimally invasive treatment. However, the choice between unilateral and bilateral transpedicular approaches remains debated due to differing surgical outcomes and complication risks. This study aims to compare the efficacy and safety of unilateral versus bilateral pedicle approaches in PKP for osteoporotic thoracolumbar vertebral compression fractures. A retrospective study was conducted at Xi’an People’s Hospital (Xi’an Fourth Hospital), including 136 patients with single-level OVCFs (T11–L2) treated with PKP between January 2021 and January 2023. Patients were grouped based on surgical approach: unilateral (n = 79) or bilateral (n = 57) pedicle puncture. Primary intraoperative outcomes included operative time, blood loss, fluoroscopic exposures, and cement volume. Secondary outcomes assessed pain relief (visual analog scale), functional recovery (activities of daily living), radiographic parameters (cement distribution, kyphotic angle, vertebral height), and complications. Baseline characteristics were similar between groups. The bilateral group had longer operative time (51.40 ± 12.99 minutes vs 45.62 ± 14.96 minutes, *P* < .05), more blood loss (15.89 ± 7.20 mL vs 9.95 ± 4.71 mL, *P* < .01), increased X-ray exposure (22.44 ± 6.15 vs 17.63 ± 6.28, *P* < .01), and greater cement volume (5.37 ± 1.64 mL vs 3.86 ± 1.41 mL, *P* < .01). Cement distribution was more extensive in the bilateral group (vertical fill height *P* < .05; horizontal fill width *P* < .01). Despite these differences, both groups experienced comparable pain relief and functional improvement (visual analog scale and activities of daily living scores, all *P* > .05). Kyphotic angle correction, vertebral height restoration, and complication rates (10.1% vs 8.8%, *P* = .50) were also comparable between the 2 groups. No cases of symptomatic cement embolism or neurological deficits were observed. Both unilateral and bilateral PKP approaches offer effective pain relief, functional recovery, and safety in treating thoracolumbar OVCFs. Although the bilateral approach achieved more symmetrical cement distribution, it required greater intraoperative resources without superior clinical outcomes. Approach selection should be individualized based on patient anatomy, fracture characteristics, and surgeon experience.

## 1. Introduction

Osteoporotic vertebral compression fractures (OVCFs) are a prevalent skeletal injury in elderly populations, often resulting from low-energy trauma or even occurring spontaneously due to weakened bone structure.^[1]^ OVCFs can lead to severe pain, spinal deformity, and reduced mobility, significantly affecting patients’ quality of life and independence. With an aging global population, the incidence of OVCFs is increasing, placing a substantial burden on healthcare systems.^[2]^ Percutaneous kyphoplasty (PKP) has emerged as an effective minimally invasive treatment for OVCFs, offering rapid pain relief, stabilization of the fractured vertebra, and improved functional outcomes.^[3]^

In PKP, inflatable balloons create a cavity in the collapsed vertebra which is then filled with polymethylmethacrylate (PMMA) bone cement, with the goals of restoring vertebral body height, correcting kyphotic deformity, and alleviating pain through mechanical stabilization.^[4]^

The choice between unilateral (single pedicle) and bilateral (both pedicles) approaches is a key technical consideration in PKP. The unilateral approach is less invasive, potentially reducing operative time and intraoperative radiation exposure for both patient and surgeon.^[5]^

A single puncture may result in less soft tissue trauma and reduced postoperative pain at the entry site. In contrast, the bilateral approach requires 2 access points, increasing procedural steps but allowing more uniform cement distribution from both sides, potentially providing better biomechanical support and reducing the risk of unfilled voids or cement bias.^[6,7]^ Indeed, finite element biomechanical studies have suggested that bilateral cement augmentation leads to more balanced stress distribution and greater vertebral stability than unilateral cement placement.^[8]^ Thus, there is a theoretical trade-off: the unilateral approach may offer a shorter, simpler surgery with less radiation and potentially fewer entry-related complications, whereas the bilateral approach could achieve optimal cement filling and possibly better long-term mechanical outcomes.^[9]^

Previous studies comparing unilateral and bilateral PKP have yielded mixed results. Some found that unilateral PKP achieves comparable clinical outcomes (pain relief and functional improvement) to bilateral PKP, while reducing operative time and fluoroscopy exposure.^[10]^

Other reports suggest that bilateral PKP might result in better radiographic restoration of vertebral height or kyphosis and reduce the likelihood of cement leakage due to the more controlled, incremental fill from 2 sides.^[11]^ Given these discrepancies in the literature,^[12]^ further investigation is warranted to guide the choice of approach.

In particular, fractures at the thoracolumbar junction (T11–L2) account for the majority of osteoporotic VCFs,^[13–15]^ due to this region’s unique biomechanical stresses and transition from rigid thoracic to mobile lumbar spine. We therefore focused our analysis on OVCFs in T11–L2 to obtain a relatively homogeneous cohort of fractures at this high-risk level. This retrospective study compares unilateral and bilateral pedicle approaches in PKP for osteoporotic fractures of T11–L2. We evaluated intraoperative parameters (surgical time, blood loss, fluoroscopy usage, cement volume), postoperative outcomes (pain relief, functional recovery, radiographic changes), and complications. Our goal was to assess whether one approach offers significant advantages in efficacy or safety, providing clinical guidance for treating OVCFs in the thoracolumbar spine.

## 2. Methods

### 2.1. Patients

This retrospective cohort study was conducted at Xi’an People’s Hospital (Xi’an Fourth Hospital) from January 2021 to January 2023. We reviewed medical records of patients with osteoporotic thoracic or lumbar vertebral compression fractures treated with PKP using either a unilateral or bilateral pedicle approach. The study was approved by the Ethics Committee (Approval No. KJLL-Z-K-2024003), and informed consent was waived due to the retrospective design.

Inclusion criteria:

(1) A single-level OVCF at the T11, T12, L1, or L2 vertebral level.
(2) Age 60 years or older.
(3) Confirmed osteoporosis (bone mineral density *T*-score ≤ –2.5).
(4) Intact posterior vertebral wall with no retropulsed bony fragments into the spinal canal.
(5) No other source of chronic back pain.

Exclusion criteria:

(1) Vertebral fractures due to malignant tumors, hemangiomas, or other non-osteoporotic pathology.
(2) Active infection at the puncture site.
(3) Signs of spinal cord compression or acute nerve injury related to the fracture.
(4) Preexisting spinal deformities or hardware that could complicate the PKP procedure.

A total of 136 patients were included in the analysis. Based on the surgical approach, patients were divided into the unilateral puncture group (n = 79) and bilateral puncture group (n = 57). In the unilateral group, PKP was performed through one pedicle on the more affected side, while in the bilateral group, PKP was performed through both pedicles.

### 2.2. Surgical procedure

All procedures were performed under local anesthesia and biplanar fluoroscopy by experienced spine surgeons. Patients were positioned prone on a radiolucent table with cushions under the chest and pelvis to achieve slight spinal hyperextension, aiding vertebral compression reduction. Gentle manual pressure and axial traction were applied before draping to realign the fractured vertebra as much as tolerated.

**Figure 1.**
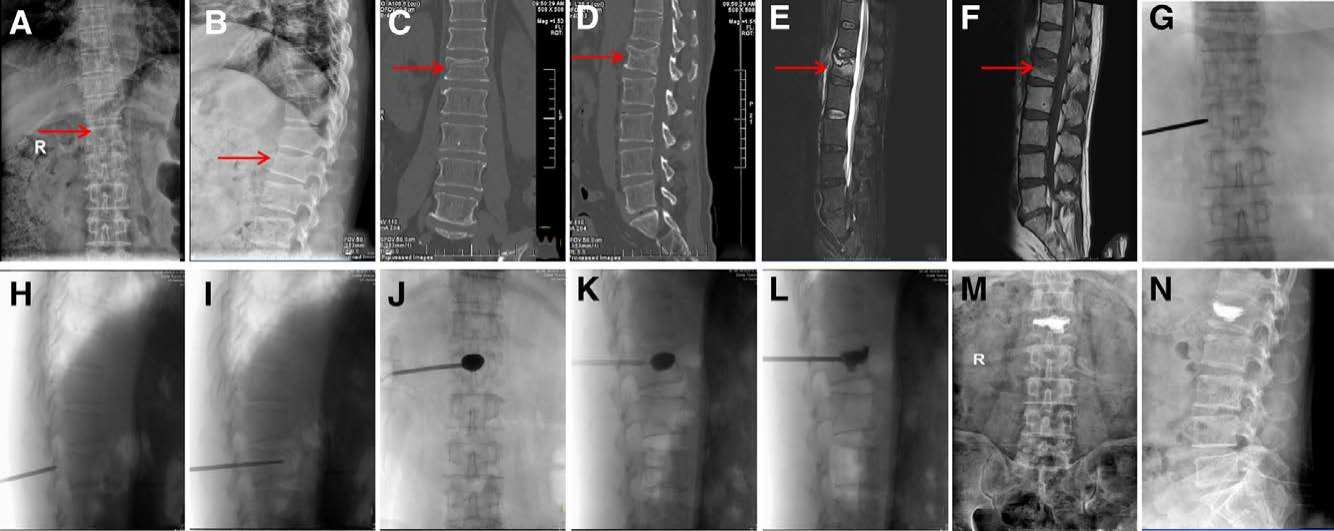
(A and B) X-ray examination shows L1 vertebral body compression. (C and D) CT scan reveals a fracture line below the superior endplate of the L1 vertebral body. (E) Sagittal MR T2-weighted image shows high signal changes (hemorrhage) at the fracture site and adjacent bone. (F) Sagittal MR T1-weighted image shows low signal changes (hemorrhage) at the fracture site and adjacent bone. (G–I) The unilateral pedicle outer wall is punctured first, followed by slow insertion of the channel into the vertebral body. A bone drill is then placed and drilled to the anterior one-third of the vertebral body. (J–L) After restoring vertebral height by balloon expansion, PMMA bone cement is slowly injected. (M and N) Postoperative follow-up X-rays (anteroposterior and lateral views) show restored vertebral height with no significant leakage of bone cement. CT = computed tomography, PMMA = polymethylmethacrylate (bone cement).

*Unilateral pedicle approach*: Under fluoroscopy, the entry point was marked 2 cm lateral to the midline, targeting the lateral margin of the pedicle on the more collapsed side. After a small incision, a bone puncture needle was advanced toward the anterior third of the vertebral body. A working cannula was placed over the needle, and a drill was used to create a cavity. A balloon tamp was inflated to restore height, then deflated and removed. PMMA cement was injected under fluoroscopic guidance until evenly distributed or extravasation was observed. In unilateral cases, the cannula was directed toward the contralateral side to promote cement flow across the midline. The procedure was completed once the cement hardened, and the incision was closed (Fig. 1).

**Figure 2.**
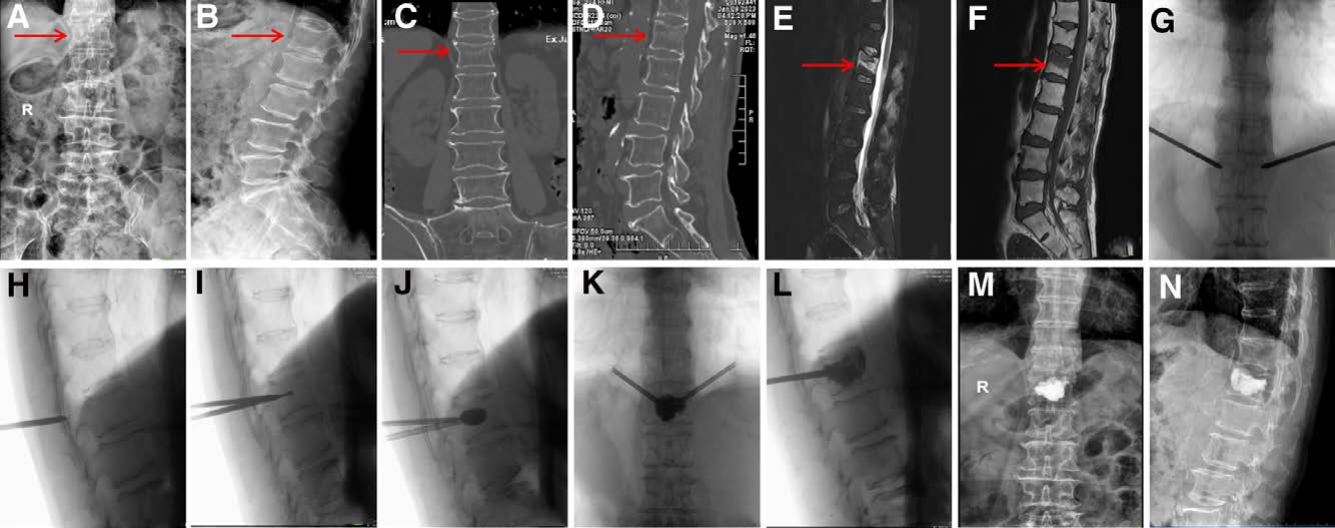
(A and B) X-ray examination suggests L1 vertebral compression; (C and D) CT scan shows a fracture line beneath the upper endplate of the L1 vertebral body; (E) MR sagittal T2-weighted image indicates high signal changes at the fracture site and adjacent bone (bleeding); (F) MR sagittal T1-weighted image indicates low signal changes at the fracture site and adjacent bone (bleeding); (G–I) first, the needle is inserted into the outer walls of both pedicles, followed by slow insertion of the channel into the vertebral body. A bone drill is then inserted to the anterior one-third of the vertebral body; (J–L) After balloon dilation to restore vertebral height, PMMA bone cement is slowly injected; (M and N) Postoperative X-ray in the anteroposterior and lateral views shows restored vertebral height with no obvious bone cement leakage. CT = computed tomography, PMMA = polymethylmethacrylate (bone cement).

*Bilateral pedicle approach*: In bilateral PKP, 2 skin entry points, 1 cm lateral to each pedicle, were marked and incised. Puncture needles were inserted into both pedicles under fluoroscopy, and working cannulas were placed after reaching the anterior third of the vertebral body. Balloon tamps were inflated to restore height, then removed. Bone cement was injected simultaneously or sequentially through both cannulas to ensure even distribution, with a larger cement volume used in bilateral cases. Injection was stopped once distribution was satisfactory or if leakage occurred. After cement hardening, both cannulas were withdrawn, and the wounds were dressed (Fig. 2).

### 2.3. Data collection

We collected data on patients’ baseline characteristics (age, sex, body mass index, fracture level, injury cause) and preoperative clinical status (baseline visual analog scale [VAS] pain score, baseline activities of daily living [ADL] score, local kyphotic angle, vertebral height). Intraoperative parameters and postoperative outcome measures were recorded as follows:

- *Operative time* (*minutes*): measured from the initiation of anesthesia to the completion of wound dressing.

*Intraoperative blood loss* (*mL*): estimated by measuring the volume in suction canisters and the change in weight of gauze swabs used (with 1 g of gauze weight increase approximating 1 mL of blood loss).^[16]^

- *Number of X-ray examinations*: the count of distinct fluoroscopic images taken (including both AP and lateral) during the procedure, as a surrogate for radiation exposure.
- *Bone cement volume* (*mL*): total volume of PMMA cement injected into the vertebral body.

Postoperative outcomes included:

- *VAS pain scores*^[17]^: recorded on a 0 to 10 visual analog scale preoperatively, and at routine follow-ups (1 month, 6 months, and 1 year post-surgery).
- *ADL scores*^[18]^: measured by a standard ADL scale (0–100, with higher scores indicating better function) preoperatively and at follow-ups (1 month, 6 months, 1 year).
- *Radiographic outcomes*: We evaluated improvement in vertebral body height (anterior height on lateral radiographs) and local kyphotic angle (Cobb angle) from preoperative to postoperative measurements. Cement distribution was assessed on postoperative radiographs, including vertical height (percentage of vertebral body height) and horizontal width (percentage of vertebral body width on AP view), to quantify cement dispersion in each group.

*Complications*: any perioperative or postoperative complications were noted, including cement leakage, new-onset neurological deficits, significant hemorrhage, infection, or refracture of the treated or adjacent vertebrae during follow-up. Clinical efficacy was also categorized by the modified MacNab criteria^[19]^ at final follow-up (excellent, good, fair, or poor, based on pain relief and functional status).

### 2.4. Statistical analysis

Statistical analyses were performed using SPSS version 26.0 (IBM Corp, Armonk). Continuous variables were presented as mean ± SD and compared using an independent-samples Student *t* test. Categorical variables were expressed as counts and percentages and compared using the chi-square test or Fisher exact test. A two-tailed *P* value < .05 was considered statistically significant.

## 3. Results

### 3.1. Baseline characteristics

A total of 136 patients with osteoporotic compression fractures at T11, T12, L1, or L2 were included, with 79 patients treated via a unilateral pedicle approach and 57 via a bilateral approach. Patient demographics and baseline clinical characteristics are summarized in Table 1. There were no significant differences between the unilateral and bilateral groups in age (*P* = .19) or sex distribution (*P* = .18). The body mass index was similar in both groups (*P* = .54). The fractured vertebral levels were comparably distributed: in the unilateral group, 21.5% T11, 29.1% T12, 35.4% L1, and 13.9% L2; in the bilateral group, 14.0% T11, 42.1% T12, 24.6% L1, and 19.3% L2 (*P* = .21). The mechanism of injury leading to fracture did not differ significantly between groups (*P* = .94). Preoperative VAS pain scores were equally high in both groups (*P* = .61), reflecting comparable pain levels before surgery. Baseline functional status (ADL score) was also similar (*P* = .43). There was no significant difference in the initial local kyphotic angle (*P* = .89) or the vertebral body height at the fracture level (*P* = .67) preoperatively.

**Table 1:** Baseline and demographic characteristics of patients.

Characteristic	Unilateral puncture group (N = 79)	Bilateral puncture group (N = 57)	*t*/*χ*^2^	*P* value
Gender			1.77	.18
Male	41 (51.90%)	23 (40.35%)		
Female	38 (48.10%)	34 (59.65%)		
Age, mean ± SD (y)	71.33 ± 8.53	73.30 ± 8.48	‐1.33	.19
BMI, mean ± SD (kg/m^2^)	22.83 ± 0.99	22.94 ± 1.12	‐0.61	.54
Fractured segments			4.49	.21
T11	17 (21.52%)	8 (14.04%)		
T12	23 (29.11%)	24 (42.11%)		
L1	28 (35.44%)	14 (24.56%)		
L2	11 (13.92%)	11 (19.30%)		
Injury mechanism, number (%)			0.12	.94
Fall	49 (62.03%)	37 (64.91%)		
Traffic accident	21 (26.58%)	14 (24.56%)		
Spontaneous	9 (11.39%)	6 (10.53%)		
Preoperative VAS, mean ± SD	6.18 ± 1.59	6.04 ± 1.66	0.5	.61
Preoperative vertebral kyphosis, mean ± SD (degree)	23.05 ± 7.21	23.20 ± 6.08	‐0.13	.89
Preoperative vertebral height, mean ± SD (mm)	18.49 ± 4.63	18.81 ± 3.93	‐0.43	.67
Preoperative ADL, mean ± SD	55.91 ± 15.04	53.89 ± 14.10	0.79	.43

ADL = activities of daily living, BMI = body mass index, L = lumbar, SD = standard deviation, T = thoracic, VAS = visual analog scale.

### 3.2. Intraoperative outcomes

**Figure 3.**
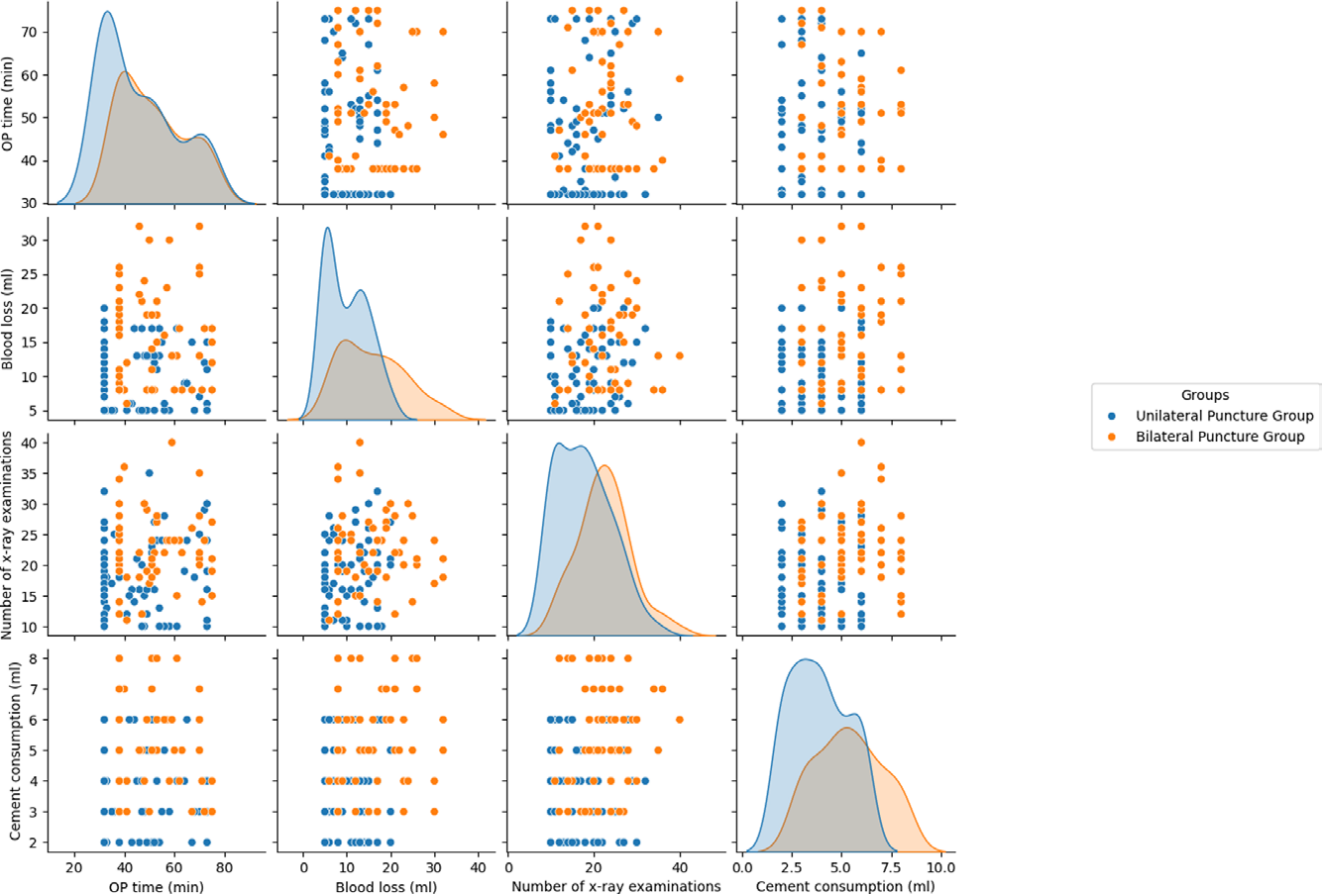
Scatter matrix of surgical outcomes. relationships between operative time, blood loss, number of X-ray examinations, and cement consumption in unilateral (blue) and bilateral (orange) puncture groups. Bilateral puncture group shows longer operative times, higher blood loss, increased X-ray examinations, and greater cement consumption.

Key intraoperative outcome measures for the 2 techniques are compared in Table 2 and illustrated in Fig. 3. The mean operative time was significantly longer in the bilateral puncture group compared to the unilateral group (51.40 ± 12.99 minutes vs 45.62 ± 14.96 minutes, *P* < .05). While both techniques resulted in minimal intraoperative blood loss, the bilateral group had slightly higher blood loss (15.89 ± 7.20 mL vs 9.95 ± 4.71 mL, *P* < .01). The unilateral approach had less bleeding, likely due to less bone and soft tissue disruption from instrumenting only one pedicle. The bilateral group also required more fluoroscopic exposures (22.44 ± 6.15 vs 17.63 ± 6.28, *P* < .01) due to the need for additional imaging when working on 2 pedicles. Finally, the bilateral group injected more bone cement (5.37 ± 1.64 mL vs 3.86 ± 1.41 mL, *P* < .01), with an average of 1.5 mL more per case due to cement being delivered from both sides to optimize fill.

**Table 2:** Comparison of intraoperative outcomes between the 2 groups.

Parameter	Unilateral puncture group (N = 79)	Bilateral puncture group (N = 57)	*t*	*P* value
OP time, mean ± SD (min)	45.62 ± 14.96	51.40 ± 12.99	‐2.35	<.05*
Blood loss, mean ± SD (mL)	9.95 ± 4.71	15.89 ± 7.20	‐5.82	<.01*
Number of X-ray examinations, mean ± SD (times)	17.63 ± 6.28	22.44 ± 6.15	‐4.44	<.01*
Cement consumption, mean ± SD (mL)	3.86 ± 1.41	5.37 ± 1.64	‐5.73	<.01*

ADL = activities of daily living, OP = operation, SD = standard deviation.

* Significant difference between the 2 groups.

### 3.3. Cement distribution and radiographic outcomes

Postoperative imaging demonstrated differences in the cement dispersion within the treated vertebrae between the 2 techniques (Table 3). In the bilateral group, bone cement covered a larger portion of the vertebral body. The mean vertical cement fill (percentage of vertebral body height) was 71.30% ± 10.28% in the bilateral group, slightly higher than 67.32% ± 10.27% in the unilateral group (*P* < .05). Similarly, the mean horizontal cement fill (percentage of vertebral body width) was significantly greater in the bilateral group (85.42% ± 8.93%) compared to the unilateral group (76.63% ± 12.11%, *P* < .01). Bilateral PKP achieved more extensive cement coverage, reaching both endplates and sides of the midline, while unilateral PKP showed more restricted cement distribution. Despite these differences, the improvement in vertebral height was comparable between groups: the unilateral group had a mean increase of 5.51 ± 4.44 mm, and the bilateral group 4.76 ± 3.25 mm (*P* = .28). Likewise, kyphotic angle correction was similar, with a mean reduction of 8.39° ± 6.82° in the unilateral group and 7.37° ± 5.10° in the bilateral group (*P* = .35).

**Table 3:** Comparative analysis of clinical outcomes between the 2 groups.

Parameter	Unilateral puncture group (N = 79)	Bilateral puncture group (N = 57)	*t*/*χ*^2^	*P* value
Vertical dispersion height of bone cement (%)	67.32 ± 10.27	71.30 ± 10.28	2.23	<.05*
Horizontal dispersion width of bone cement (%)	76.63 ± 12.11	85.42 ± 8.93	4.64	<.01*
Vertebral kyphosis improvement, mean ± SD, (degree)	8.39 ± 6.82	7.37 ± 5.10	0.95	.35
Vertebral height improvement, mean ± SD, (mm)	5.51 ± 4.44	4.76 ± 3.25	1.09	.28
VAS, mean ± SD				
Pre-OP	6.18 ± 1.59	6.04 ± 1.66	0.51	.61
1 month post-OP	1.08 ± 0.97	1.25 ± 1.11	0.95	.35
6 months Post-OP	0.78 ± 0.73	0.89 ± 0.78	0.83	.41
1 year post-OP	0.57 ± 0.64	0.59 ± 0.57	‐0.2	.85
ADL, mean ± SD				
Pre-OP	55.91 ± 15.04	53.89 ± 14.10	0.79	.43
1 month post-OP	75.46 ± 15.92	76.93 ± 15.22	0.54	.59
6 months post-OP	80.18 ± 12.97	80.02 ± 12.81	0.07	.94
1 year post-OP	83.32 ± 10.09	83.94 ± 11.40	0.33	.74
Modified MacNab criteria			2.55	.47
Excellence	39 (49.37%)	24 (42.11%)		
Good	32 (40.51%)	28 (49.12%)		
Fair	6 (7.59%)	2 (3.51%)		
Poor	2 (2.53%)	3 (5.26%)		
Clinical efficacy classification			0.07	.79
Excellence and good	71 (89.87%)	52 (91.23)		
Fair and poor	8 (10.13%)	5 (8.77%)		
Complications			2.34	.50
Cementum extravasation	4 (57.14%)	3 (42.86%)		
Hemotoma	0 (0%)	1 (14.29%)		
Neurological injury	1 (14.29%)	0 (0%)		
Refracture	2 (28.57%)	3 (42.86%)		

ADL = activity of daily living, OP = operative, pre-OP/post-OP = preoperative/postoperative, SD = standard deviation, VAS = visual analog scale.

* Indicates that the difference was statistically significant.

### 3.4. Pain and functional outcomes

**Figure 4.**
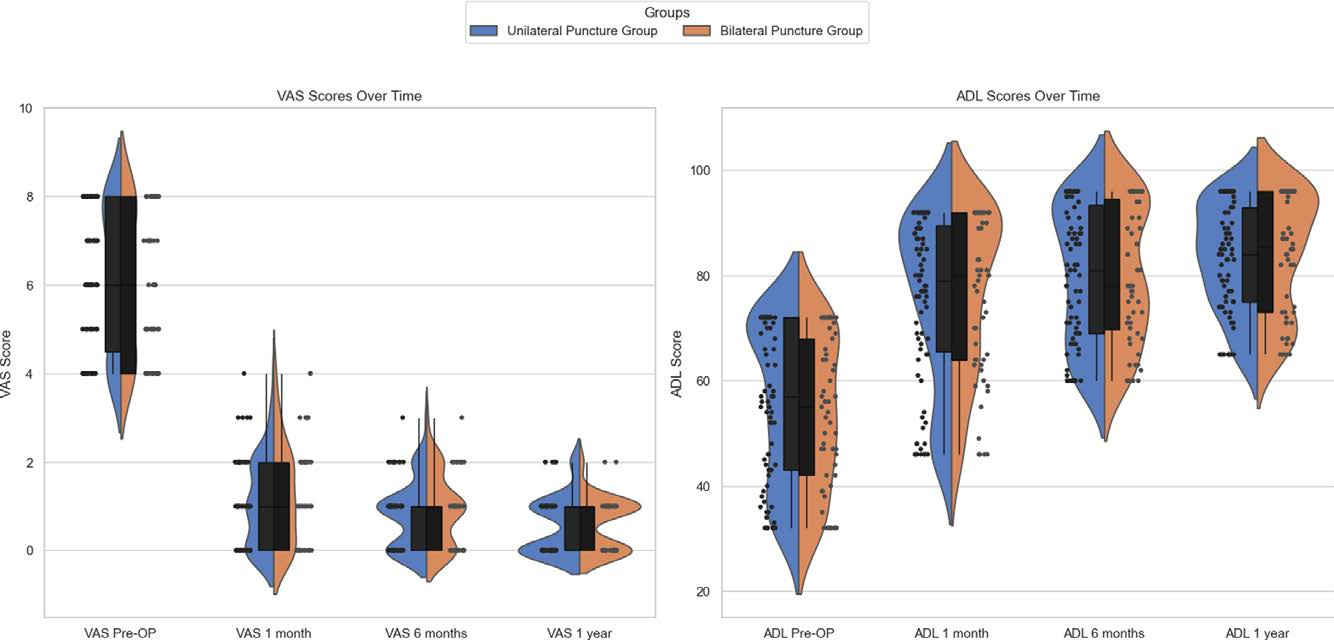
Distribution of VAS and ADL scores over time. comparison of VAS and ADL scores over time between unilateral puncture group and bilateral puncture group. The plots combine violin plots, box plots, and jittered data points to provide a detailed view of the data distribution and individual observations. ADL = activities of daily living, VAS = visual analog scale.

Both approaches provided excellent pain relief and functional gains, with no statistically significant differences in clinical outcomes over the follow-up period (Table 3 and Fig. 4).

Preoperatively, patients in both groups reported severe pain and impaired daily function. At 1 month postoperatively, VAS scores improved significantly, with the unilateral group reporting 1.1 ± 1.0 and the bilateral group 1.3 ± 1.1 (*P* = .35). By 6 months, scores improved further to 0.8 (unilateral) versus 0.9 (bilateral) (*P* = .41), and at 1 year, the VAS was 0.57 (unilateral) versus 0.59 (bilateral) (*P* = .85). The pain reduction trajectory was similar, with no significant differences between the 2 approaches.

In terms of functional recovery, ADL scores improved substantially for both groups. The unilateral group’s mean ADL increased from 55.9 preoperatively to 75.5 at 1 month, 80.2 at 6 months, and 83.3 at 1 year. The bilateral group showed similar improvement: from 53.9 preoperatively to 76.9 at 1 month, 80.0 at 6 months, and 83.9 at 1 year. No significant differences were observed at any time point (e.g., 1-year ADL: 83.32 vs 83.94, *P* = .74). The overlap in violin/box plots of VAS and ADL between groups (Fig. 4) highlights the comparable clinical efficacy of the 2 approaches in patient outcomes.

### 3.5. Clinical efficacy and complications

Clinical efficacy, as assessed by the modified MacNab criteria at final follow-up, was high in both groups with no significant difference (Table 3). In the Unilateral group, 89.9% of patients had “excellent” or “good” outcomes, compared to 91.2% in the Bilateral group (*P* = .79). Only a few patients in each group had “fair” or “poor” outcomes, with no significant differences.

The overall complication rates were low and similar between the 2 approaches (10.1% in the unilateral group vs 8.8% in the bilateral group, *χ*^²^ = 2.34, *P* = .50). The most common complication was minor cement leakage. Asymptomatic cement extravasation occurred in 4 patients (5.1%) in the unilateral group and 3 patients (5.3%) in the bilateral group, with no clinical symptoms or need for intervention. Neurological complications were rare: 1 case of transient intercostal nerve irritation in the unilateral group (resolving spontaneously in 2 weeks) and no nerve injuries in the bilateral group. One patient (1.8%) in the bilateral group developed a small paravertebral muscle hematoma, managed conservatively. Refractures occurred in 2 patients (2.5%) in the unilateral group and 3 (5.3%) in the bilateral group, reflecting underlying osteoporotic risk rather than procedural complications. No cases of pulmonary cement embolism, deep infection, or other severe complications were reported.

## 4. Discussion

OVCFs in the thoracolumbar region can cause debilitating pain and deformity in elderly patients.^[20]^ PKP has become a widely accepted minimally invasive treatment to quickly stabilize these fractures and alleviate pain.^[21]^ This study directly compares unilateral and bilateral PKP techniques for treating osteoporotic compression fractures at T11–L2. Both approaches were highly effective in pain relief and functional recovery, with comparable safety profiles. However, we observed differences in intraoperative parameters and cement distribution outcomes between the 2 techniques.

As expected, the bilateral pedicle approach resulted in longer operative times and greater resource utilization due to the need to cannulate 2 pedicles. On average, bilateral PKP took about 6 minutes longer than unilateral PKP. This aligns with previous reports that unilateral PKP tends to shorten surgical time by eliminating the second pedicle cannulation.^[22,23]^ The bilateral group required approximately 5 more fluoroscopic images on average, reflecting the need for more extensive instrument maneuvering on both sides. From a radiation safety standpoint, the unilateral approach can reduce cumulative X-ray exposure for the patient and surgical team.^[24,25]^ Blood loss was minimal for both techniques, with unilateral cases showing slightly less bleeding (by 6 mL). Although the difference is small and clinically insignificant, it suggests that a single puncture creates a smaller access tract, resulting in less bleeding.

As expected, bilateral PKP used approximately 1.5 mL more cement than unilateral PKP. By approaching from both sides, cement fills the vertebra more evenly, achieving broader coverage. Our measures confirmed that bilateral injections resulted in greater vertical and horizontal cement spread within the vertebral body. A more extensive cement fill may enhance biomechanical support. Biomechanical analyses, including a finite element study by Dai et al, suggest that bilateral cement augmentation better balances internal stresses and improves vertebral stability compared to unilateral, potentially reducing the risk of collapse under load.^[8]^ In our clinical data, however, there were no significant differences in immediate postoperative height restoration or kyphosis correction between the 2 groups. Both approaches restored vertebral height by 4 to 6 mm and reduced the local kyphotic angle by 7° to 8°, with no advantage for bilateral PKP. It appears that the balloon tamp step primarily determines height restoration, and as long as adequate cement is injected to stabilize this height, final kyphosis correction is similar regardless of the number of cement injections.^[26]^ The more homogeneous cement filling achieved with bilateral PKP may improve long-term vertebral strength and durability. Some studies^[27]^ suggest it could reduce the risk of recompression or adjacent level fractures, though our data showed no significant difference in 1-year refracture rates.

Clinical outcomes were similar between the unilateral and bilateral groups at 1-year follow-up. Both techniques provided rapid and sustained pain relief, with VAS scores dropping to 1 or below after 1 month and remaining low. This reduction highlights the effectiveness of vertebral augmentation in stabilizing fracture site micro-movements and alleviating pain. The absence of differences in VAS scores at all postoperative intervals aligns with previous studies and meta-analyses, which have shown comparable pain improvement with either unilateral or bilateral kyphoplasty.^[28]^ Functional recovery, as measured by ADL scores and MacNab criteria, was excellent and independent of the approach. Both groups regained high daily function by 6 to 12 months, with nearly 90% rated excellent/good. This indicates that unilateral PKP is as effective as bilateral PKP in restoring quality of life after an OVCF.

Both techniques exhibited low complication rates when performed by experienced surgeons. No symptomatic cement pulmonary embolism or neurologic injuries requiring intervention were observed. The rates of asymptomatic cement leakage (5% in both groups) are in line with reported rates for kyphoplasty, which are generally lower than those for vertebroplasty due to the use of the balloon cavity.^[29]^ According to a meta-analysis by Chen W,^[30]^ the unilateral approach did not increase the risk of cement leakage on the contralateral or posterior side, and the bilateral approach did not completely eliminate this risk, which is consistent with our observed results. A single case of transient intercostal neuralgia in the unilateral group likely resulted from the needle trajectory at the thoracic level and resolved without issue. No such events occurred in the bilateral group, possibly due to fewer upper thoracic levels. The incidence of new vertebral fractures was small and similar between groups. Although literature debates whether increased cement volume affects adjacent vertebrae, our data showed no significant difference in adjacent fractures within 1 year.

*Technical considerations*: The unilateral approach requires precise needle placement to ensure cement reaches the opposite side of the vertebra. In our practice, we angle the needle medially to achieve sufficient cement dispersion across the midline in the thoracolumbar region. However, narrow pedicles or asymmetric fractures may limit unilateral efficacy, making bilateral access preferable for complete fill. Approach selection was based on surgeon preference and intraoperative judgment. If unilateral injection failed to adequately fill the vertebra, a second pedicle was occasionally cannulated, placing the case in the bilateral group. Conversely, challenging anatomy or difficult contralateral access led to a unilateral approach to minimize operative time and radiation exposure. Overall, the unilateral approach is sufficient for most single-level OVCFs, offering shorter procedures and less radiation, while bilateral approaches are reserved for cases requiring maximal cement spread.

This study has several limitations due to its retrospective design. The choice of unilateral versus bilateral approach was made by the treating surgeons based on clinical judgment, potentially introducing selection bias. We minimized this by matching baseline characteristics and focusing on a narrow range of fracture levels (T11–L2). The follow-up duration was limited to 1 year, which may not capture long-term outcomes like kyphosis correction durability or new fractures. Additionally, our cement distribution measures are semiquantitative and based on plain radiographs; advanced imaging or cadaveric studies would be needed for more precise assessment. Despite these limitations, our study benefits from a reasonable sample size for a single-center series and reflects real-world surgical outcomes.

A prospective, randomized controlled trial would provide a definitive comparison between unilateral and bilateral PKP, minimizing selection bias. Longer follow-up is needed to determine if clinical outcomes diverge over time or if one approach offers more sustained benefits. Additionally, investigating patient subsets, such as fractures with severe comminution or specific morphologies, could identify situations where one technique may be superior. Evaluating adjacent level degeneration or fracture rates in a larger cohort over time could clarify the impact of cement volume or distribution.

## 5. Conclusion

In summary, both unilateral and bilateral transpedicular approaches in PKP are effective for treating osteoporotic compression fractures at the thoracolumbar junction. The unilateral approach offers shorter operation time, less radiation exposure, and reduced cement usage, while the bilateral approach provides more homogeneous cement distribution. Our study found no significant differences in pain relief, functional recovery, radiographic restoration, or complication rates between the 2 methods up to 1 year postoperatively. Both approaches offer excellent clinical outcomes and are safe and reliable. The choice of approach should be based on patient anatomy, fracture pattern, surgeon experience, and intraoperative findings. Unilateral PKP is a time-efficient option for straightforward fractures, while bilateral PKP may be preferred for complex fractures or when maximal cement coverage is needed. Tailoring the surgical strategy to each patient ensures optimal balance between efficiency and biomechanical reinforcement.

## Acknowledgments

The authors have no acknowledgments to declare (no third-party assistance was used in the writing of this manuscript).

## Author contributions

**Data curation:** Haigang Zhang, Xiaoliang Ma.

**Formal analysis:** Zhiyu Liu, Bin Shi.

**Funding acquisition:** Zhiyu Liu.

**Investigation:** Zhiyu Liu, Haigang Zhang, Xiaoliang Ma, Weiliang Zhang, Bin Shi.

**Methodology:** Zhiyu Liu, Haigang Zhang, Xiaoliang Ma.

**Project administration:** Zhiyu Liu, Weiliang Zhang, Bin Shi.

**Resources:** Haigang Zhang, Xiaoliang Ma.

**Supervision:** Bin Shi.

**Validation:** Zhiyu Liu, Bin Shi.

**Visualization:** Zhiyu Liu, Weiliang Zhang, Bin Shi.

**Writing – original draft:** Haigang Zhang, Weiliang Zhang.

**Writing – review & editing:** Bin Shi.
